# Store-operated calcium channels in skin

**DOI:** 10.3389/fphys.2022.1033528

**Published:** 2022-10-05

**Authors:** Declan Manning, Caroline Dart, Richard L Evans

**Affiliations:** ^1^ Institute of Systems, Molecular and Integrative Biology, University of Liverpool, Liverpool, United Kingdom; ^2^ Department of Physiology and Membrane Biology, School of Medicine, University of California, Davis, Davis, CA, United States; ^3^ Unilever Research and Development, Port Sunlight Laboratory, Bebington, Wirral, United Kingdom

**Keywords:** skin, store-operated calcium entry, STIM, Orai channels, TRPC channels, keratinocytes, melanocytes, eccrine sweat gland

## Abstract

The skin is a complex organ that acts as a protective layer against the external environment. It protects the internal tissues from harmful agents, dehydration, ultraviolet radiation and physical injury as well as conferring thermoregulatory control, sensation, immunological surveillance and various biochemical functions. The diverse cell types that make up the skin include 1) keratinocytes, which form the bulk of the protective outer layer; 2) melanocytes, which protect the body from ultraviolet radiation by secreting the pigment melanin; and 3) cells that form the secretory appendages: eccrine and apocrine sweat glands, and the sebaceous gland. Emerging evidence suggests that store-operated Ca^2+^ entry (SOCE), whereby depletion of intracellular Ca^2+^ stores triggers Ca^2+^ influx across the plasma membrane, is central to the normal physiology of these cells and thus skin function. Numerous skin pathologies including dermatitis, anhidrotic ectodermal dysplasia, hyperhidrosis, hair loss and cancer are now linked to dysfunction in SOCE proteins. Principal amongst these are the stromal interaction molecules (STIMs) that sense Ca^2+^ depletion and Orai channels that mediate Ca^2+^ influx. In this review, the roles of STIM, Orai and other store-operated channels are discussed in the context of keratinocyte differentiation, melanogenesis, and eccrine sweat secretion. We explore not only STIM1-Orai1 as drivers of SOCE, but also independent actions of STIM, and emerging signal cascades stemming from their activities. Roles are discussed for the elusive transient receptor potential canonical channel (TRPC) complex in keratinocytes, Orai channels in Ca^2+^-cyclic AMP signal crosstalk in melanocytes, and Orai isoforms in eccrine sweat gland secretion.

## Introduction

Skin forms the largest organ system in the human body, and is comprised of a vast layer of dermal and epidermal tissues which protect the body from the surrounding environment. Ca^2+^ signals are utilised throughout the skin to control the functions of a range of cell types, including keratinocytes, melanocytes, eccrine sweat glands and cells from the immune and nervous systems. Each adopt a unique Ca^2+^ signalling toolkit to support their specific cellular functions. In recent decades, store-operated Ca^2+^ entry (SOCE), whereby depletion of intracellular Ca^2+^ stores triggers Ca^2+^ influx across the plasma membrane, has emerged as a critical process regulating differentiation, melanogenesis and sweat secretion. SOCE dysfunction in skin is linked to anhidrotic ectodermal dysplasia, hyperhidrosis, dermatitis, hair loss and cancer (Gwack et al., 2008; Stanisz et al., 2016; Lian et al., 2017; Evans et al., 2018; Yeh et al., 2020). To initiate SOCE, store-resident stromal interaction molecules (STIMs) sense Ca^2+^ depletion, cluster and activate plasma membrane store-operated Ca^2+^ (SOC) channels (Numaga-Tomita & Putney, 2013; Liou et al., 2005; Zhang et al., 2005). While the different cells of the skin and its appendages express several SOC isoforms, the predominant channels in this process are believed to be members of the Orai family, and principally Orai1 (Bovell et al., 2015, 2016; Lian et al., 2017; Yeh et al., 2020). Several transient receptor potential (TRP) proteins are also proposed to act as SOCs and TRP dysfunction is linked to numerous skin pathologies (Leuner et al., 2011; Caterina and Pang, 2016). This review will discuss the essential roles of STIM1 and Orai1 in keratinocyte, melanocyte and eccrine sweat gland function, as well as the emerging role for Orai1-driven TRP canonical channel (TRPC) activity in keratinocyte differentiation.

## Keratinocytes: Differentiation

**FIGURE 1:**
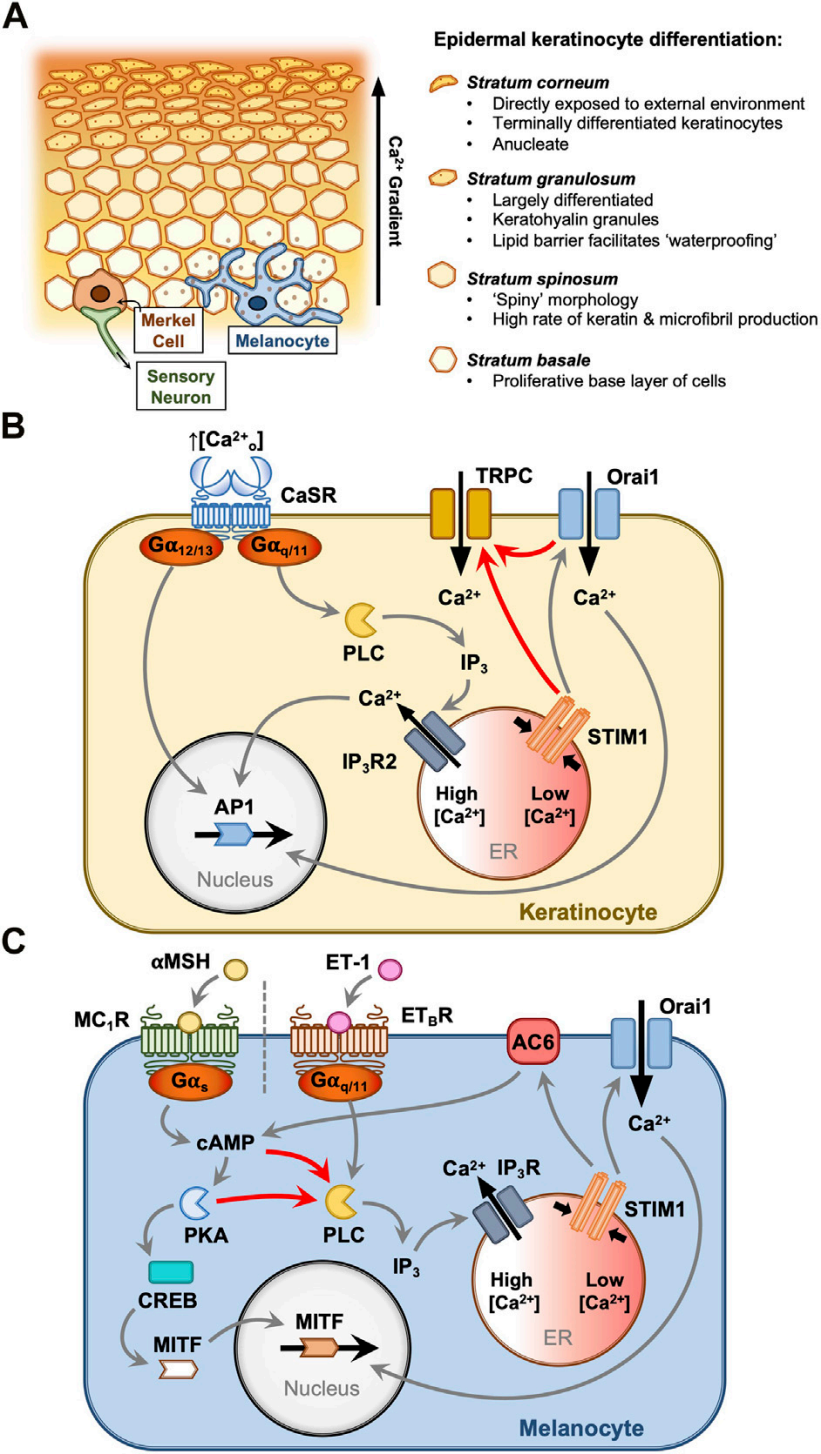
STIM1 and Orai1 drive keratinocyte and melanocyte functions in the epidermis. Keratinocytes and melanocytes are critical protective cells in the epidermal layer **(A)**. Keratinocytes proliferate in the basal layer and differentiate in response to elevated extracellular [Ca^2+^]. This is driven by the Ca^2+^-sensing receptor (CaSR) **(B)**, which activates Gα_12/13_ and Gα_q/11_ proteins. ER Ca^2+^ mobilisation triggers SOCE, and the resultant cytosolic Ca^2+^ signals contribute to AP1 activation and differentiation **(B)**. Large currents carried by TRPC channels accompany the Orai channel Ca^2+^ influx, although the mechanism of TRPC channel activation in keratinocytes is not understood. Melanocytes protect the body from ultraviolet radiation by secreting melanin granules into the surrounding tissue. This is controlled by two key pathways **(C)**, each triggered by paracrine hormones secreted from the surrounding cells **(C)**. α-Melanocyte stimulating hormone (αMSH) and endothelin-1 (ET-1) stimulate melanocyte-inducing transcription factor (MITF)-driven melanogenesis via Gα_s_ and Gα_q/11_ pathways, respectively. Both pathways prompt IP_3_-mediated ER Ca^2+^ store depletion and SOCE. Store depletion resulting from αMSH activity causes direct activation of adenylyl cyclase (AC6) by STIM1, producing a positive feedback loop where cyclic AMP production drives either protein kinase A (PKA) or EPAC activation to further activate phospholipase C (PLC), Ca^2+^ store depletion and SOCE.

Keratinocytes form approximately 95% of all cells in the epidermis. Here, these cells differentiate to form a protective ‘cornified’ outer layer (Figure 1A). This process is tightly controlled by the transcriptional factor family activator protein-1 (AP1; Eckert et al., 1997). As cells differentiate, they largely lose their proliferative capacity, express keratinocyte-specific differentiation markers (such as involucrin), and form intercellular desmosome linkages which confer mechanical strength to the outer skin layer (Capone et al., 2000). An epidermal Ca^2+^ gradient, rising towards the surface layers, and maintained primarily *via* the sequestration of intracellular Ca^2+^ in cells of the stratum granulosum (Celli et al., 2016), drives the keratinocyte differentiation process (Tu et al., 2005). Cells detect elevated extracellular Ca^2+^ concentration ([Ca^2+^]_o_) by the Ca^2+^-sensing receptor (CaSR), a promiscuous G-protein coupled receptor that couples to several pathways (Figure 1B) including phospholipase C (PLC) activation and hence IP_3_ generation, store depletion and SOCE (Hofer and Brown, 2003; Tu et al., 2005).

In the HaCaT keratinocyte cell line, increasing the [Ca^2+^] in the culture media from 0.03 to 1.8 mM triggers a switch away from the proliferative phenotype that the cells adopt under low-confluence conditions *in vitro*. This ‘Ca^2+^ switch’ induces expression of keratin 1, which acts to slow cell growth (Tu et al., 2005). Knockdown of either STIM1 or Orai1 has been shown to ablate inward Ca^2+^ release-activated Ca^2+^ current (I_CRAC_) and reduce keratin one expression when cells are switched to 1.8 mM Ca_o_^2+^. Additionally, HaCaT cells lacking STIM1 or Orai1 exhibit diminished proliferative responses to changes in Ca_o_^2+^(Numaga-Tomita & Putney, 2013; Takei et al., 2016). SOCE via Orai1 has been shown to govern a range of functions in primary keratinocytes including proliferation, differentiation and migration (Vandenberghe et al., 2013) and Orai1 knockout mice consistently display a thin-skinned phenotype (Gwack et al., 2008). Ca^2+^ entry through STIM1-activated Orai1 channels thus seems vital for the routine functions of keratinocytes.

TRPC channels were first implicated in the keratinocyte SOC response in a study with primary human keratinocytes (Tu et al., 2005). Using siRNA knockdowns of TRPC1 and TRPC4, Tu and colleagues used Ca^2+^ imaging to demonstrate the involvement of these channels in keratinocyte SOCE. They also identified a physical interaction between TRPC1, IP_3_ receptors (IP_3_Rs) and PLCγ1 (Tu et al., 2005). TRPC1 and TRPC4 involvement was later confirmed in separate studies with primary human keratinocytes from gingiva (Cai et al., 2006; Fatherazi et al., 2007) and neonatal foreskin (Beck et al., 2008). Fatherazi et al. (2007) examined the biphasic SOC response with patch-clamp electrophysiology, highlighting an initial inward-rectifying current characteristic of I_CRAC_, followed by an ohmic current more characteristic of heteromeric TRPC channels. TRPC1, C5, C6 and C7 mRNA and protein were initially identified in gingival keratinocytes (Cai et al., 2005) compared with TRPC1, C4, C5 and C7 in HaCaT keratinocytes (Beck et al., 2006). Müller and colleagues demonstrated that specific activation of TRPC6 with hyperforin stimulated differentiation of HaCaT keratinocytes (Müller et al., 2008). Hence, there is uncertainty over the specific combinations of TRPC isoforms involved in keratinocyte SOC currents. Furthermore, the specific mechanism of action for this differentiation trigger event requires further clarification, with no link between Orai and TRPC channel activity identified in keratinocytes.

The consequences of impaired Ca^2+^ signalling in keratinocytes are also unclear. One group has demonstrated reduced TRPC1-7 expression and store-operated Ca^2+^ influx in primary keratinocytes harvested from psoriatic patients (Leuner et al., 2011). Celli et al. (2021) also demonstrated a reduction in store-operated Ca^2+^ influx in aged primary human keratinocytes, alongside reduced CaSR and PLCβ/γ expression, and impaired cell-to-cell adhesion. Recent studies in mice have also suggested that STIM1 may act as a primary thermo-sensor in keratinocytes *via* heat-induced regulation of Orai channels (Nwonkonko et al., 2019). However, it remains unclear whether STIM1 impacts thermosensation in human skin. Keratinocytes additionally play an essential role in superficial wound repair, and much evidence suggests that impaired SOCE processes would inhibit their ability to proliferate and repair damaged tissue (Tu et al., 2005; Darbellay et al., 2014; Numaga-Tomita & Putney, 2013). However, one *in vivo* study demonstrated that STIM1 knockout actually improves skin injury outcomes in mice (Putney et al., 2017). It is postulated that this is due to pro-inflammatory chemokine release from keratinocytes, which subsequently boosts protective neutrophil activity (Su and Richmond, 2015). Additional studies are required to resolve the contradictory evidence concerning SOCE processes in wound repair.

## Melanocytes: Melanogenesis and proliferation

Melanocytes distribute melanin pigment among neighbouring keratinocytes to defend the immediate tissue from mutation and damage resulting from ultraviolet radiation (Figure 1A). The literature indicates that this skin pigmentation process is regulated by two different pathways (Figure 1C). One pathway facilitates ‘adaptive tanning’ in response to environmental ultraviolet radiation (UV) exposure (Stanisz et al., 2012; Nam & Lee, 2016; Kawakami & Fisher, 2017), and another pathway is linked more directly with baseline melanin production while also contributing to adaptive melanogenesis (Chakraborty et al., 1996; Scott et al., 2002; Cui et al., 2007). In this section, the emerging role of STIM and Orai will be discussed for each of these pathways.

Melanin production is induced by the activation of melanocyte-inducing transcription factor (also known as microphthalmia-associated transcription factor, MITF; Hodgkinson et al., 1993). MITF controls melanocyte differentiation, metabolism, proliferation and survival through its regulation of a range of genes. The most relevant in melanogenesis is its directed upregulation of tyrosinase (Tyr), which initiates the biosynthetic pathway for melanin (Kuzumaki et al., 1993). Both of the melanogenesis pathways described here converge on the upregulation of MITF in melanocytes, which is consistently found to drive skin pigmentation and melanocyte proliferation (reviewed by Kawakami & Fisher, 2017).

One distinct melanin production pathway is stimulated by α-melanocyte stimulating hormone (αMSH), and to a lesser extent, adrenocorticotropic hormone (ACTH), both of which are produced by neighbouring keratinocytes by cleaving proopiomelanocortin (POMC) (Chakraborty et al., 1996). αMSH activates melanocortin one receptor (MC_1_R) on nearby melanocytes to stimulate melanogenesis and proliferation through both Gα_s_ signalling via cyclic AMP (cAMP) and Gβγ signalling via MAPK pathway activation (Cui et al., 2007). Point mutations in the POMC and MC_1_R genes can each produce a red-haired, fair-skinned phenotype characterised by low baseline melanin production (Valverde et al., 1995; Krude et al., 1998). Thus, αMSH-MC_1_R signalling is a factor in setting ‘baseline’ melanin production in the epidermis. Furthermore, UV radiation causes keratinocytes to increase production of αMSH, and elevated αMSH levels also stimulate melanocytes to upregulate MC_1_R expression (Chakraborty et al., 1996; Scott et al., 2002; Cui et al., 2007). As such, the αMSH-MC_1_R pathway appears capable of contributing to baseline melanin production and also stimulating an adaptive response to UV radiation (Cui et al., 2007).

cAMP has long been considered critical in the αMSH melanogenesis pathway for its role in activating PKA, which in turn activates nuclear cAMP response element-binding protein (CREB) which then activates MITF (D’Orazio et al., 2006) (Figure 1C). The role of cAMP signalling in melanocyte responses to αMSH is well characterised, however a role for Ca^2+^-dependent signalling proteins has only recently been proposed for this signalling network. Motiani et al. (2018) characterised SOCE in primary human melanocytes, B16 melanoma cells and a zebrafish melanogenesis model. Primary human melanocytes with greater melanin content were shown to undergo enhanced SOCE (Motiani et al., 2018). αMSH and the adenylyl cyclase (AC) activator, forskolin, each stimulated endoplasmic reticulum (ER) Ca^2+^ mobilisation, and Ca^2+^ release could be blocked by inhibitors of either AC or PLC, interlinking PLC activity and Ca^2+^ store depletion with the cAMP production caused by MC_1_R activation (Motiani et al., 2018). It is unclear whether cAMP indirectly activates PLC in these cells via phosphorylation events induced by PKA or via signalling through exchange protein directly activated by cAMP (EPAC). Orai1 knockdown in both primary melanocytes and B16 cell tumours inhibited αMSH-stimulated proliferation (Motiani et al., 2018). STIM1 knockdown also reduced melanocyte and melanoma growth, and furthermore knockdown of zebrafish STIM1a significantly reduced *in vivo* melanin production (Motiani et al., 2018). This result was mirrored in B16 melanoma cells where, surprisingly, STIM1 knockdown reduced melanin production but Orai1 knockdown had no effect (Motiani et al., 2018). Intriguingly, it was shown that this effect was exerted by direct activation of AC6 by STIM1, which interacts via the serine/proline-rich domain (SPD), generating cAMP (Motiani et al., 2018). Taken together, these data suggest a positive feedback loop whereby MC_1_R-generated cAMP causes ER Ca^2+^ store depletion and STIM1 oligomerisation, and independent of Orai1, STIM1 generates cAMP through AC6 activation to amplify the cAMP melanogenesis signal (Motiani et al., 2018).

When exposed to UV radiation, keratinocytes in the epidermis release several hormones to influence the growth and behaviour of neighbouring melanocytes. While one melanogenesis pathway is known to be activated by αMSH, locally-released endothelin-1 (ET-1) activates the melanocyte endothelin B receptor (ET_B_R) to trigger a distinct pathway in response to UV exposure (Kawakami & Fisher, 2017). ET_B_R stimulation activates PLC to generate diacylglycerol (DAG) and IP_3_-mediated Ca^2+^ store mobilisation, which concurrently stimulate the RAS/MAPK signalling pathway to drive MITF activation and melanogenesis (Imokawa et al., 1996; Sato-Jin et al., 2008) (Figure 1C). Stanisz et al. (2012) showed that Orai1 knockdown blunts *in vitro* primary melanocyte viability, and inhibition with 2-APB inhibits tyrosinase activity and UV- or ET-1-induced melanin production. This demonstrated that Orai1 activation and SOCE play a critical role in adaptive melanogenesis, specifically in response to ET-1 signalling. This was supported by Nam & Lee (2016), who showed that Orai channel inhibition with trans-anethole reduces UV-induced melanin production in B16 melanoma cells. Surprisingly, this study found that tyrosinase activity was preserved despite Orai1 inhibition, suggesting that SOCE might bypass MITF to upregulate melanin production in B16 cells (Nam & Lee, 2016). Thus, the precise role of Orai1 channels in ET-1-induced melanogenesis requires further clarification.

The role of STIM1 and Orai1 in melanocyte biology has been detailed by relatively few studies, which may be explained by the fact that melanocyte research is often linked to the goal of understanding melanoma pathology (reviewed by Stanisz et al., 2016 and Zhang et al., 2022). Studies have reported differing Orai1-3 and STIM1-2 expression profiles across model systems, and although STIM2 expression often exceeds that of STIM1, it appears to be STIM1 that activates Orai channels and AC6 in melanocytes (Stanisz et al., 2012, 2014; Motiani et al., 2018). Furthermore, the reported effects of STIM and Orai activity on melanocyte proliferation, migration, invasion and survival vary considerably between studies and according to the model system studied (Stanisz et al., 2012, 2014; Sun et al., 2014; Hooper et al., 2015).

## The eccrine sweat gland: ‘Clear’ cell secretion

**FIGURE 2:**
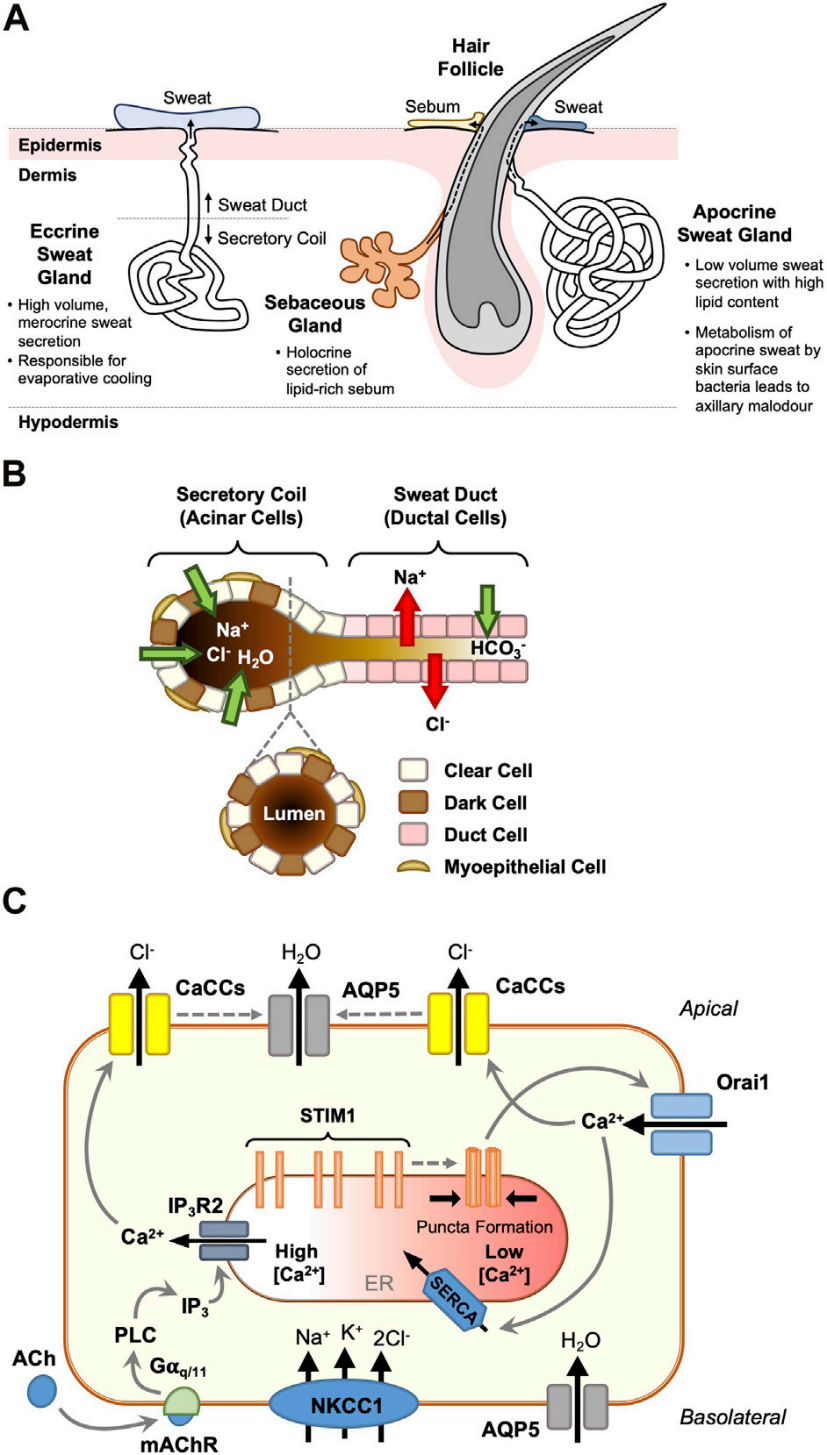
STIM1 and Orai1 are critical regulators of eccrine clear cell sweat secretion. Distinct from the apocrine sweat gland and the sebaceous gland which each associate with hair follicles **(A)**, the eccrine sweat gland secretes dilute sweat directly onto the skin surface. It is responsible for salt and fluid secretion, through a two-phase merocrine secretory mechanism **(B)**. Acinar cells in the secretory coil drive fluid secretion by actively transporting Cl^−^, with Na^+^ following across the tight junctions, and water osmotically. Myoepithelial cells provide structural support to the secretory cells, whilst upstream ductal cells modify the ionic composition of the hypertonic primary sweat, ready for release onto the skin surface. Clear cells are the key drivers of eccrine fluid secretion **(C)**. In addition the transepithelial transport of Cl^−^ ions (via the basolateral Na^+^-K^+^-2Cl^-^ co-transporter, NKCC1, and Ca^2+^-activated Cl^−^ channels, TMEM16A and Best2), these cells move water from the plasma to the gland lumen via basolateral and apical aquaporin 5 (AQP5) channels in response to stimulation from the sympathetic nerve termini (*via* mAChR activation). Gα_q/11_ proteins generate a biphasic cytosolic Ca^2+^ signal through sequential IP_3_-mediated ER Ca^2+^ release and store depletion resulting in STIM1-mediated Orai1 activation and SOCE. Elevated cytosolic [Ca^2+^] activates apical Ca^2+^-activated Cl^−^ channels (CaCCs) and Cl^−^ efflux, resulting in osmotically-driven water efflux into the sweat gland lumen **(C)**.

Human skin contains three secretory appendages: the eccrine and apocrine sweat glands, and the sebaceous gland. The former facilitates thermoregulation by secreting a clear salty fluid (sweat) directly onto the skin surface, whilst the latter two secrete more viscous lipid- and protein-containing secretions (apocrine sweat and sebum respectively) into the hair follicular space (Figure 2A). The processes involved in eccrine gland sweat production are well understood and have been reviewed extensively (Sato et al., 1989a, 1989b; Wilke et al., 2007; Bovell, 2018; Cui et al., 2018). In brief, in eccrine sweat gland secretory coil cells (notably the so-called ‘clear cells’, but also the ‘dark’ cells), sweating is initiated via a two-step process: an agonist-generated rise in [Ca^2+^]_i_ (the ‘Ca^2+^ signal’), followed by the activation of Cl^−^ transport across the epithelium from the plasma to the glandular lumen (‘Cl^−^ transport’; Figure 2B). The principal agonist initiating thermoregulatory sweating is acetylcholine, with adrenaline and other catecholamines controlling stress-induced emotional sweating.

The ion transport proteins involved in the Cl^−^ transport process have been characterised at the molecular level, but it is only recently that insights into the protein components involved in generating the Ca^2+^ signal have been reported. Cl^−^ influx is driven by the basolateral Na^+^-K^+^-2Cl^-^ co-transporter NKCC1 (Zhang et al., 2014), and Cl^−^ efflux via the Ca^2+^-activated Cl^−^ channels (CaCCs) TMEM16A (Anoctamin-1) and bestrophin 2 (Ertongur-Fauth et al., 2014; Wilson and Metzler-Wilson, 2015; Concepcion et al., 2016) (see Figure 2C). The water channel aquaporin 5 (AQP5) is present in the apical membrane (Zhang et al., 2014) and knock-out studies in mice have suggested a central role for this channel in fluid secretion (Nejsum et al., 2002).

A key event in the generation of the ‘Ca^2+^ signal’ that triggers eccrine sweating is a biphasic increase in the level of [Ca^2+^]_i_ comprising an initial agonist-induced release of Ca^2+^ from internal stores, followed by an influx of extracellular Ca^2+^. This SOCE process is widely documented in many exocrine cells (see Ambudkar, 2015; Concepcion & Feske, 2017). The first indication that SOCE in human eccrine gland cells may involve Ca^2+^-sensing STIM proteins and Ca^2+^-selective Orai channels was reported in the eccrine gland NCL-SG3 cell line (Bovell et al., 2015). Subsequent studies using freshly isolated human eccrine secretory coil cells showed that STIM1, Orai1 and Orai3 are expressed at both gene and protein levels (Bovell et al., 2016). Immunofluorescence demonstrated that STIM1 is located intracellularly (probably in the ER), and Orai one and three in the plasma membrane of secretory coil cells. Fluorescence imaging experiments showed that influx of extracellular Ca^2+^, triggered by the emptying of intracellular stores with thapsigargin, was perturbed using a variety of SOCE inhibitors (carboxyamidotriazole, diethylstilbesterol and BTP2; Bovell et al., 2016). Furthermore, thapsigargin-induced SOCE was reduced by ∼50% in an Orai1 knockdown, whilst Orai3 knockdown was ineffective. This suggested that with respect to SOCE, Orai3 can partially substitute for Orai1, and Orai1 can totally substitute for Orai3 in respective knockdowns (Bovell et al., 2016). A double Orai1-Orai3 knockdown nearly abolished SOCE. These data suggest that STIM1 and Orai1 are the principal drivers of SOCE and hence Cl^−^ flux and sweat secretion in eccrine secretory coil cells, with Orai3 providing a supportive role which remains undefined. The suggestion that STIM1, and not STIM2, is the Ca^2+^ sensor critical for sweat secretion is supported by a mouse study, which shows that foot pad sweat secretion is significantly inhibited in a STIM1 knockout but is comparable to the wild type in a STIM2 knockout (Concepcion and Feske, 2017).

The significance of Orai channels in sweat gland function is further highlighted in human patients suffering loss-of-function mutations in their STIM1 and Orai1 genes, which results in anhidrosis (an inability to produce eccrine sweat) and hyperthermia at high ambient temperatures due to an intrinsic incapacity for evaporative cooling (Concepcion et al., 2016). Furthermore, NCL-SG3 cells lacking STIM1 and Orai1 expression are characterised by a loss of SOCE functionality and agonist-induced Cl^−^ secretion *via* TMEM16A (Concepcion et al., 2016).

Finally, a study using freshly isolated eccrine secretory coil cells from patients suffering from hyperhidrosis (sweating that exceeds that required for normal body temperature regulation), showed that the [Ca^2+^]_i_ response following stimulation with the muscarinic agonist carbachol was 2-fold that of cells isolated from non-hyperhidrotic individuals (Evans et al., 2018). Expression of STIM1 in hyperhidrotic cells was also double that of non-hyperhidrotics, while expression of Orai1, the muscarinic m3 receptor, IP_3_ isoforms 1 and 2, and AQP5 were not significantly changed. Over-expression of STIM1 at the protein level was confirmed by Western blotting and immunohistochemistry, whilst STIM2 was not over-expressed (Evans et al., 2018). Thapsigargin-induced SOCE was reduced in STIM1 and Orai1 knockdowns. These observations suggested that human hyperhidrotic eccrine gland secretory coil cells are characterised by an over-expression of STIM1 relative to non-hyperhidrotic individuals, resulting in enhanced SOCE, which presumably leads to higher sweat secretion.

## Conclusion

Numerous skin cell types rely on Ca^2+^ entry channels to control their routine physiological functions. Here, the roles of SOCE channels have been summarised for a selection of key skin functions: keratinocyte differentiation, melanocyte proliferation and melanogenesis, and sweat secretion from ‘clear’ cells in the eccrine sweat gland. This selection is by no means exhaustive, with cells from the immune and nervous systems resident in the integumentary system playing vital roles governed by their own unique Ca^2+^ entry systems. STIM1 and Orai1 have been revealed as key drivers of all the skin functions discussed here, both through their well-characterised SOCE process, the independent actions of STIM1, and the uncharacterised signal cascades stemming from their activities. Clearly, much is yet to be determined about the role of non-Orai store-operated Ca^2+^ channels such as the elusive TRPC complex in keratinocytes, the role of Orai channels in Ca^2+^-cAMP signal crosstalk in melanocytes, and the interaction of different Orai isoforms in driving eccrine sweat gland fluid secretion.
